# Design and Production of Respirable Effervescent Microparticles to Enhance Drug Penetration Through Lung Mucus

**DOI:** 10.3390/pharmaceutics18070837

**Published:** 2026-07-09

**Authors:** Valentina Ruggiero, Francesca Mariano, Domenico Larobina, Gaetano D’Avino, Marco Trofa, Giovanni Falcone, Pasquale Del Gaudio, Paola Russo

**Affiliations:** 1 Department of Pharmacy, University of Salerno, Via Giovanni Paolo II, 132, 84084 Fisciano, Italy; vruggiero@unisa.it (V.R.); fmariano@unisa.it (F.M.); gifalcone@unisa.it (G.F.); pdelgaudio@unisa.it (P.D.G.); 2 Institute of Polymers, Composites and Biomaterials, National Research Council of Italy, P.le E. Fermi, 1, 80055 Portici, Italy; domenico.larobina@cnr.it; 3 Dipartimento di Ingegneria Chimica, dei Materiali e della Produzione Industriale, Università degli Studi di Napoli Federico II, P. le Tecchio 80, 80125 Naples, Italy; gadavino@unina.it (G.D.); marco.trofa@unina.it (M.T.)

**Keywords:** inhalable microparticles, spray drying, effervescence, pulmonary drug delivery, mucus barrier, particle engineering, triple-fluid nozzle

## Abstract

**Background/Objectives**: Dry powder inhalation (DPI) is a promising strategy for the treatment of respiratory diseases such as cystic fibrosis (CF), where thick and viscous mucus limits drug penetration and contributes to persistent infection and inflammation. Although inhalation allows rapid drug action with reduced systemic exposure, its efficacy depends on the ability of inhaled drugs to achieve and maintain therapeutic concentrations in the lungs and to overcome airway barriers. This study aimed to develop and characterize effervescent dry powder formulations designed to enhance mucus permeabilization through mechanical disruption while delivering an antibiotic. **Methods**: Effervescent microparticles containing sodium bicarbonate, an organic acid (citric or tartaric acid), and levofloxacin were produced by spray drying using a triple-fluid nozzle to control component distribution and prevent premature effervescence. The influence of functional excipients, including L-leucine and mannitol, on particle formation, aerosol performance, and process yield was evaluated. Microparticles were characterized in terms of morphology, fine particle fraction (FPF), and effervescence-related properties. **Results**: Formulations containing L-leucine and citric acid reduced particle agglomeration and achieved a fine particle fraction of up to approximately 18%, although with a lower process yield. In contrast, formulations based on tartaric acid and mannitol improved both production yield and aerosol performance, with FPF values increasing up to 27.3% and more efficient CO_2_ release. The resulting microparticles exhibited spherical, hollow, and partially fragmented morphology, consistent with premature CO_2_ generation during spray drying. **Conclusions**: The effervescent approach, combined with controlled spray drying parameters, represents a promising formulation strategy to modulate particle behavior and drug release in mucus-relevant environments. These findings support further investigation of effervescent DPI systems for improved pulmonary drug delivery in CF.

## 1. Introduction

Cystic fibrosis (CF) is a genetic disorder characterized by the accumulation of highly viscous mucus within the airways, which promotes chronic bacterial infections and significantly hinders drug transport [1]. Despite advances in antibiotic therapy, effective treatment of pulmonary infections remains challenging due to the limited penetration of drugs across the mucus barrier, which restricts access to the underlying epithelium and infection sites.

Inhalation therapy using dry powder inhalers (DPIs) represents an attractive strategy for localized drug delivery to the lungs, offering high local concentrations while minimizing systemic exposure [2,3]. However, the performance of inhaled powders in CF is strongly influenced by the interaction between particles and mucus, which can impair particle dispersion, slow drug release, and limit transport through the airway surface liquid [4]. Consequently, formulation strategies capable of modulating particle behavior in mucus-relevant environments are of considerable interest.

Several approaches have been studied to overcome the pulmonary mucus barrier. Chemical and biochemical strategies dominate current research, including: (i) mucolytic agents (e.g., N-acetylcysteine, dornase alfa) that directly degrade mucus components [5,6]; (ii) mucus-penetrating particles engineered with low-density coatings or specific surface chemistries to minimize adhesion [7,8]; and (iii) combination therapies that simultaneously deliver drugs and mucolytic agents [1].

While promising, these approaches face significant limitations in the CF context: mucolytic therapies may show variable efficacy depending on the heterogeneous biochemical composition and viscoelastic properties of CF mucus, and excessive mucus disruption may negatively affect mucociliary clearance mechanisms [6]. Similarly, conventional nanoparticles can become trapped within the mucus mesh and undergo rapid mucociliary clearance, limiting their residence time and drug bioavailability in distal lung regions [9].

In contrast, physical approaches aimed at transiently modulating mucus rheology through mechanical rather than biochemical interactions remain underexplored and may represent a complementary and potentially more robust alternative to conventional muco-active therapies, particularly by reducing the dependence on the highly variable biochemical composition of CF mucus.

Effervescence-based systems, widely employed in oral dosage forms to enhance disintegration and dissolution, generate gas upon contact with aqueous media, producing localized mechanical stress and fluid convection. Moreover, in effervescent suppositories, the release of carbon dioxide gas creates gentle pressure that distends the rectal walls. This mechanical stimulus triggers the defecation reflex, naturally prompting the bowel muscles to contract and move stool along [10]. Translating this concept to inhalable dry powders, a weak and localized effervescence could offer a purely physical mechanism to improve drug dissolution and transiently perturb viscous mucus without relying on biochemical interactions, which can be influenced by the highly heterogeneous and patient-specific composition of CF mucus.

In this context, spray drying, particularly using multi-fluid nozzles, provides a versatile platform to engineer composite microparticles with controlled composition and internal architecture [11]. By spatially separating reactive components within the same particle, it is possible to prevent premature reactions during processing while enabling weak gas generation upon contact with biological fluids.

The aim of this study was therefore to investigate an effervescent formulation strategy based on multi-fluid spray drying to produce inhalable levofloxacin-containing microparticles capable of generating a controlled and weak effervescence upon hydration. The influence of formulation composition and excipient selection on particle morphology, powder properties, aerosol performance, and drug release behavior under mucus-relevant conditions was systematically evaluated.

## 2. Materials and Methods

### 2.1. Materials

Sodium bicarbonate (Bic), L-Leucine (L-Leu), and Mannitol (Mann) were obtained from Sigma-Aldrich (Milan, Italy). Anhydrous citric acid (Cit) and anhydrous tartaric acid (Tart) were purchased from AppliChem GmbH (Damstadt, Germany). Levofloxacin (LVX) was kindly donated by Genetic SpA (Fisciano, Italy).

Clear, colorless size 3 gelatin capsules were purchased from Farmalabor (Canosa di Puglia, Italy). The Monodose DPI RS01 model 7 inhaler device was kindly supplied by Plastiape SPA (Lecco, Italy). Purified water was obtained by reverse osmosis using a Milli-Q system (Millipore, Molsheim, France). For dissolution/permeation experiments, mucus was collected from porcine gastric mucosa obtained from a local slaughterhouse. Briefly, porcine gastric mucus (PGM) was isolated from freshly excised pig stomachs and used without further purification. The stomachs were opened longitudinally, and the internal surface was carefully rinsed with deionized water to eliminate residual debris. Portions of approximately 10 × 10 cm^2^ were excised and stored frozen until use. Prior to each experiment, a tissue section was thawed at room temperature, and the mucus layer was gently recovered from the mucosal surface using a spatula.

### 2.2. Microparticle Preparation

Microparticles were produced using spray drying technology with a Mini Spray Dryer B-290 (Buchi Laboratoriums-Tecnik, Flawil, Switzerland) equipped with a triple-fluid nozzle. In this configuration, organic acids were introduced through the outer channel of the nozzle, while sodium bicarbonate and levofloxacin were delivered via the inner channel.

Sodium bicarbonate and citric acid were employed at a 3:1 molar ratio, consistent with the stoichiometry of the CO_2_-generating reaction, at concentrations of 3.00% *w*/*v* and 2.50% *w*/*v*, respectively. Both compounds were dissolved in distilled water until complete solubilization. Initial formulations were prepared using only citric acid and sodium bicarbonate.

For formulations based on tartaric acid, sodium bicarbonate and tartaric acid were used at concentrations of 3.00% *w*/*v* and 2.68% *w*/*v*, respectively, in accordance with the stoichiometry of the effervescent reaction, in which one mole of tartaric acid reacts with two moles of sodium bicarbonate. Both components were dissolved in distilled water until complete solubilization. Initial formulations were prepared using only tartaric acid and sodium bicarbonate.

Depending on the formulation, L-leucine and Mannitol were added at 5.0% or 10.0% w/w relative to the mass of drug in the corresponding feed. In some batches, they were incorporated into both feed solutions, while in others, it was added only to the outer feed as a dispersing agent to improve powder flowability and reduce particle aggregation. Subsequently, levofloxacin (1.50% *w*/*v*) was incorporated into the inner solution.

The spray parameters were set as follows: inlet temperature 120–130 °C; drying air flow 500 L/min; aspiration rate 28–35 m^3^/h; air pressure 7 atm. Two different feed rates were evaluated by synchronizing two separate peristaltic pumps. The outer feed was delivered at a higher flow rate (3.0 mL·min^−1^) than the inner feed (2.0 mL·min^−1^). The outlet temperature varied between 72 °C and 78 °C depending on the composition of the feed fluids (Table 1).

All spray-dried powders were collected and stored at room temperature.

### 2.3. Levofloxacin Quantification

Levofloxacin content in spray-dried powders, as well as samples collected during in vitro aerodynamic assessment, was determined by UV spectrophotometry (Evolution 201, Thermo Fisher Scientific, Ozzano dell’Emilia, Bologna, Italy) at 287 nm using a 1 cm SUPRASIL^®^ quartz cuvette (Hellma 100-QS, HELLMA Italia srl, Milan, Italy). The analytical procedure was validated using aqueous standard solutions of levofloxacin over the concentration range of 1.83–18.3 μg/mL (y = 0.0758x + 0.0298; R^2^ = 0.9942). For release experiments, levofloxacin quantification was carried out at 331 nm using the same quartz cuvette. In this case, calibration curves were prepared in 0.05 M phosphate buffer (pH 7.4) within the concentration interval of 6.43–51.4 μg/mL (y = 0.0317x + 0.0006; R^2^ = 0.9999).

### 2.4. Spray Drying Yield

After the powder was obtained using the Spray Dryer, it was collected and stored in glass vials at room temperature. Each batch was weighed to determine the percentage yield of the process using the following formula:

(1) $$Yield\;(\%)=\frac{mass\;of\;recovered\;powder\;(g)}{mass\;of\;powder\;in\;the\;liquid\;feed\;(g)}\times 100$$

### 2.5. Particle Size

Particle size distribution of the microparticles was evaluated by laser light scattering using a granulometer equipped with a Tornado dry powder dispersion module (LS 13 320, Beckman Coulter Inc., Miami, FL, USA). The instrument operates with a 5 mW laser diode at a wavelength of 750 nm and incorporates reverse Fourier optics combined with a fiber-optic spatial filter and binocular lens system. Particle size analysis was performed using the instrument software (Beckman Coulter LS, version 5.01) based on the Fraunhofer optical model. The Tornado dispersion system allows dry powder analysis under conditions comparable to wet dispersion methods, while avoiding the use of solvents that could potentially modify particle surface characteristics [12]. Samples were loaded into a plastic cylinder to achieve an obscuration value ranging from 4% to 8%. Results were reported as median particle diameter (d50) and span, calculated according to the equation: span = [d(90) − d(10)]/d(50), where d(10), d(50), and d(90) correspond to the particle diameters at the 10th, 50th, and 90th percentiles of the particle size distribution, respectively.

### 2.6. Microparticle Morphology

Microparticle morphology was investigated by scanning electron microscopy (SEM) using a Zeiss EVO MA10 instrument equipped with a secondary electron detector (Carl Zeiss SMT AG, München-Hallbergmoos, Germany). Analyses were carried out at an accelerating voltage of 14 kV. Prior to observation, samples were coated with a gold layer approximately 200–440 Å thick using a Leica EMSCD005 sputter coater (Leica Microsystems, Wetzlar, Germany) [13].

### 2.7. Bulk, Tapped, True Density and Carr’s Index

Bulk density (ρb) and tapped density (ρt) of the spray-dried powders were determined according to a previously reported procedure [14]. Briefly, powder samples were transferred into a bottom-sealed 1 mL plastic syringe (Terumo Europe, Leuven, Belgium), which was then closed with laboratory film (Parafilm^®^ “M”, Pechiney Plastic Packaging, Chicago, IL, USA). The syringe was repeatedly tapped against a hard surface until no further reduction in powder volume was observed. As a custom syringe-based method was employed instead of the standard pharmacopeial procedure, the resulting Carr’s Index values should not be directly compared with official reference limits. Nevertheless, the method provides internally consistent measurements suitable for comparative evaluation among the investigated formulations.

Bulk and tapped densities were calculated as the ratio between the net powder mass and the corresponding powder volume before and after tapping, respectively. Carr’s Index was calculated according to Equation (2).

(2) $$Carr’s\;index\;(\%)=\frac{\rho_{t}-\rho_{b}}{\rho_{t}}\times 100$$

Experiments were performed in triplicate.

### 2.8. Aerodynamic Properties

The aerodynamic performance of the spray-dried powders was assessed in vitro using a Single Stage Glass Impinger (SSGI, Copley Scientific Ltd., Nottingham, UK) coupled with a Monodose DPI RS01 model 7 inhaler device (Plastiape S.p.A., Lecco, Italy). The lower and upper chambers of the SSGI were filled with 30 mL and 7 mL of water, respectively. Clear, colorless size 3 gelatin capsules were manually filled with 15–30 mg (±0.5 mg) of microparticles, depending on powder density. Prior to testing, each capsule was inserted into the inhaler and pierced twice to facilitate powder emission. Deposition experiments were carried out using a vacuum pump (ERWEKA GmbH, Heusenstamm, Germany) operating at a flow rate of 60 (±5) L/min for 5 s. Following aerosolization, deposited material was recovered by rinsing each chamber of the SSGI with water for subsequent quantification. The emitted dose (ED) was determined gravimetrically and expressed as the percentage of powder released from the device relative to the amount initially loaded into the capsule. The Fine Particle Fraction (FPF) was expressed as the percentage of drug associated with particles having an aerodynamic diameter below 6.4 μm, corresponding to the fraction collected in the lower chamber relative to the total drug loaded into the capsules.

### 2.9. In Vitro Drug Release

**Figure 1:**
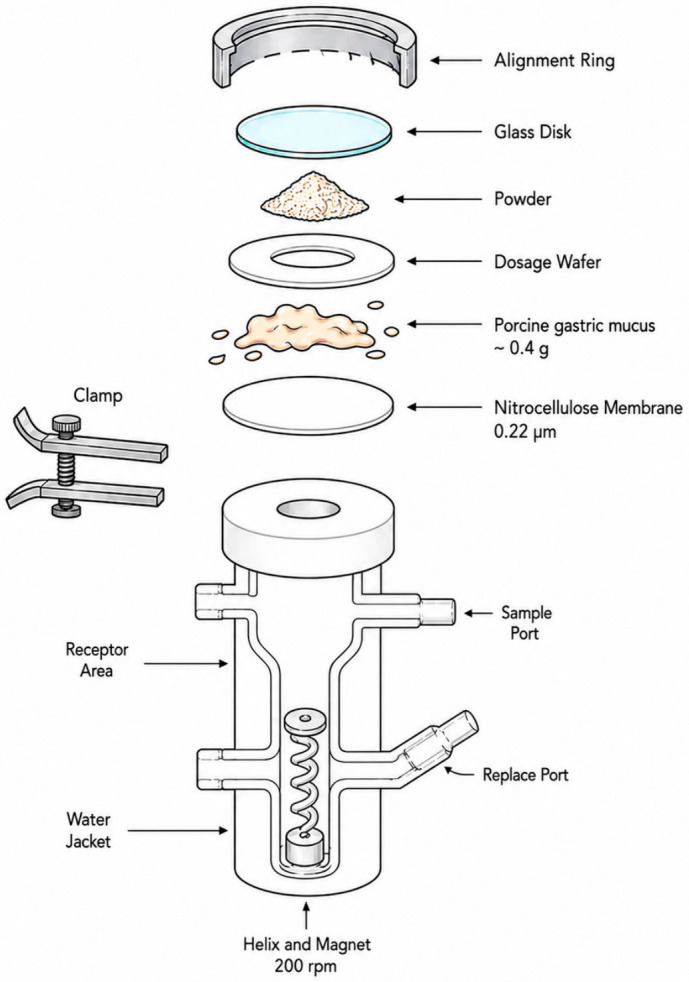
Schematic representation of the Franz diffusion cell assembled in a vertical configuration. A layer of porcine gastric mucus is applied between the membrane and the powder to mimic a mucosal barrier. Adapted from [15] and refined using AI-assisted image editing software ChatGPT (OpenAI, San Francisco, CA, USA; GPT-4-based model).

In vitro drug release and diffusion through mucus were evaluated using Franz-type vertical diffusion cells (Hanson Research Corporation, Chatsworth, CA, USA). The system temperature was maintained at 37 °C throughout the experiments by circulating water from a thermostatically controlled bath, while continuous stirring at 200 rpm was ensured using Teflon-coated magnetic bars placed in the receptor compartment. Initial experiments were performed using a standard Franz cell configuration, with the powder deposited directly onto the membrane surface. The receptor chamber was filled with 7 mL of 0.05 M phosphate buffer (pH 7.4), and a nitrocellulose membrane (0.22 μm pore size, GSWP02500, Merck Millipore Ltd., Billerica, MA, USA), previously equilibrated with the same buffer, was positioned between donor and receptor compartments, providing an effective diffusion area of 1.77 cm^2^. Approximately 5 mg of untreated levofloxacin was accurately weighed and placed onto the membrane within the donor compartment. For formulation testing, an amount of powder equivalent to 5 mg of levofloxacin, calculated according to the drug content of each formulation, was applied. The donor compartment was then sealed using spring clips and laboratory film (Parafilm^®^). In a second experimental setup, a thin layer (1.5 mm) of porcine gastric mucus was interposed between the nitrocellulose membrane and the applied formulation (Figure 1). At predetermined time intervals (15, 30, 60, 120, 180, 240, 300, 360, and 420 min), aliquots of 500 μL were withdrawn from the receptor compartment and replaced with an equal volume of prewarmed buffer. Levofloxacin concentration was determined by UV spectrophotometry (Evolution 201, Thermo Fisher Scientific, Ozzano dell’Emilia, Bologna, Italy) at 331 nm.

## 3. Results and Discussions

### 3.1. Dry Powders Production and Characterization

The aim of this work was to develop levofloxacin-loaded inhalable microparticles designed to improve local pulmonary drug delivery in the presence of an altered mucus barrier, a pathological condition that limits therapeutic efficacy in CF. To overcome this limitation, sodium bicarbonate and organic acid were selected as functional excipients to enable an in situ effervescent reaction upon contact with mucus. The generation of carbon dioxide (CO_2_), resulting in a mild and localized effervescence, is expected to induce transient physical perturbations within the mucus matrix, potentially influencing levofloxacin transport. The main technological challenge in the development of in situ effervescent microparticles was the optimization of the spray drying process to ensure particle formation while preventing premature reaction between sodium bicarbonate and organic acid. For this purpose, a spray dryer equipped with a triple-fluid nozzle was selected. This configuration allows separate feeding of sodium bicarbonate and the organic acid in distinct solutions, minimizing their contact prior to atomization and reducing the risk of premature effervescence.

Citric acid was initially selected due to its widespread use in effervescent formulations and its well-established safety [16]. Sodium bicarbonate and citric acid were separately dissolved in distilled water at 3.0% *w*/*v* and 2.5% *w*/*v*, respectively, according to the stoichiometry of the CO_2_-generating reaction:

C_6_H_8_O_7_ + 3NaHCO_3_ → Na_3_C_6_H_5_O_7_ + 3H_2_O + 3CO_2_

To better understand the influence of the acid component, tartaric acid was investigated as an alternative organic acid, as it has been reported to have lower hygroscopicity than citric acid [17]. Also in this case, the concentrations were defined to ensure the stoichiometric production of CO_2_:

C_4_H_6_O_6_ + 2NaHCO_3_ → Na_2_C_4_H_4_O_6_ + 2H_2_O + 2CO_2_

In particular, tartaric acid and sodium bicarbonate were dissolved separately at concentrations of 2.68% *w*/*w* and 3.00% *w*/*w*, respectively.

The experimental work initially focused on the production of batches composed exclusively of organic acid and sodium bicarbonate, namely CB batches containing citric acid and TB batches containing tartaric acid.

Spray drying was performed at an inlet temperature of 120 °C and an aspiration rate of 28 m^3^/h. These conditions were chosen to increase particle residence time and promote efficient moisture removal, considering the hygroscopic nature of both components.

However, the resulting powders exhibited pronounced hygroscopicity and poor handling properties, highlighting the need to introduce excipients capable of improving particle properties and reducing aggregation without compromising the effervescent mechanism.

Two different excipients were therefore investigated: L-leucine and mannitol. L-leucine was selected for its well-known ability to migrate to the droplet surface during spray drying, forming a hydrophobic layer that reduces interparticle cohesive forces and limits moisture penetration [18,19]. Mannitol, on the other hand, was chosen for its favorable physicochemical properties. Its crystalline solid state promotes the formation of spherical particles, which are generally associated with improved powder flowability and more reproducible aerodynamic performance [20,21].

Moreover, mannitol is particularly advantageous as it is less hygroscopic than other sugars and does not absorb moisture until relative humidity exceeds 90%. This property contributes to the enhanced physical and chemical stability of DPI formulations [22,23,24]. In the specific context of CF, its use provides an additional therapeutic advantage. Mannitol has been extensively investigated as an inhaled agent capable of inducing an osmotic gradient within the airways, promoting water influx into the bronchial lumen. This mechanism enhances hydration of the airway surface liquid and improves mucociliary clearance [25].

Both excipients were incorporated at 10% *w*/*w* relative to the mass of drug in the corresponding feed, while keeping the operating conditions unchanged, to evaluate their impact on process performance and on the characteristics of the resulting microparticles. Spray drying yields and particle size distributions were markedly influenced by formulation composition and excipient selection (Table 2).

Overall, formulations containing only the effervescent components (CB and TB batches) exhibited moderate to high yields (≈52–54%), indicating stable atomization and efficient particle recovery under the selected process conditions. A reduction in yield was observed for batches containing L-leucine, independent of the type of acid used in the effervescent system (CB_LEU10 and TB_LEU10). This behavior can be attributed to the surface enrichment of L-leucine during spray drying, which promotes the formation of highly porous particles [26]. Although L-leucine forms a hydrophobic outer layer that improves powder flowability and reduces interparticle cohesion, the resulting particles are more easily entrained within the drying chamber and cyclone, leading to increased powder loss and lower recovery.

In contrast, the inclusion of mannitol led to a partial recovery of yield, likely due to its tendency to crystallize during drying, which promotes the formation of denser and less adhesive particles, thereby improving collection efficiency. The higher temperature promoted more efficient solvent evaporation, while the increased aspiration reduced particle residence time, thereby limiting particle-wall interactions. As a result, TB_MAN10c achieved the highest yield among the mannitol-containing batches (41.1%), likely reflecting the formation of denser and more compact particles with reduced adhesion to the drying chamber surfaces.

For drug-loaded batches, levofloxacin was incorporated into the inner feed along with sodium bicarbonate, while L-leucine or mannitol remained in the outer feed. The inclusion of the active ingredient did not significantly affect the yield trends previously observed for the carrier-based systems.

Particle size analysis of the drug-loaded batches revealed that the presence and concentration of excipient influenced the median diameter (D_50_), a critical parameter for pulmonary deposition. In L-leucine-containing batches, increasing the leucine content in the outer feed led to a concentration-dependent reduction in median diameter (D_50_), from 7.66 µm for CB_LVX_LEU5 to 6.28 µm for CB_LVX_LEU10. This effect is likely due to the surfactant-like properties of L-leucine, which lower surface tension during atomization, producing smaller droplets and consequently smaller spray-dried particles [27]. The smallest particles were obtained for formulations combining leucine and levofloxacin (e.g., CB_LVX_LEU10), suggesting a synergistic effect on atomization and particle formation. Formulations containing mannitol (TB_MAN series) tended to exhibit larger particle sizes and, in some cases, broader distributions, likely due to differences in solute crystallization and droplet solidification mechanisms.

The SPAN values indicated moderate-to-broad particle size distributions across all batches. Notably, TB-based formulations exhibited higher SPAN values than CB-based formulations, suggesting greater heterogeneity in particle formation, possibly due to the different physicochemical properties of tartaric- and citric-acid-based systems.

In particular, the lower steric hindrance associated with tartaric acid may facilitate interactions with all the other formulation components during the drying process, thereby contributing to increased variability in particle size distribution [28,29].

The presence of leucine did not consistently reduce the SPAN values, indicating that although leucine influences particle morphology and mean particle size, its effect on distribution uniformity is less pronounced. Overall, these findings highlight the key role of formulation composition, particularly the inclusion of excipients such as L-leucine and mannitol, in governing both process yield and particle size characteristics. This behavior likely reflects the complex interplay among droplet composition, drying kinetics, and particle formation mechanisms during spray drying.

Another critical parameter influencing pulmonary deposition is particle morphology, including shape and surface characteristics, as these directly affect powder dispersibility, interparticle cohesion, and aerodynamic behavior.

**Figure 2:**
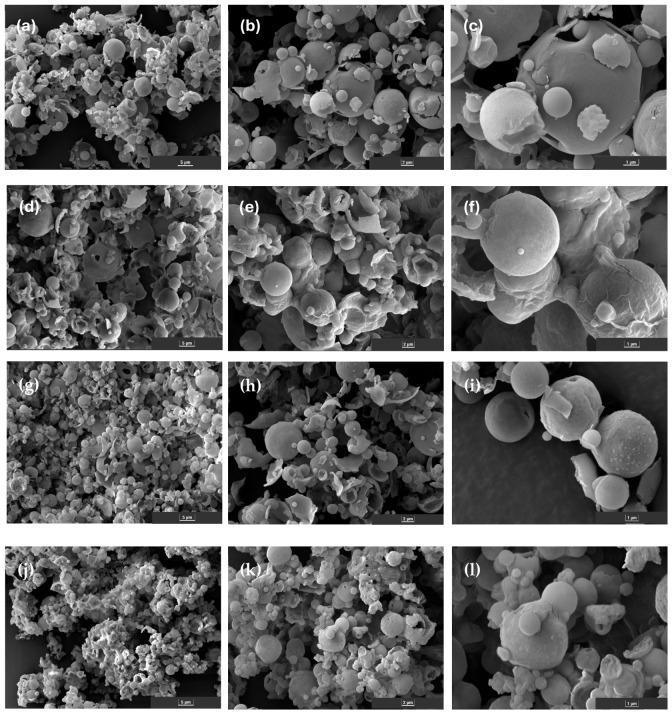
SEM images of formulations CB_LVX_LEU10 (**a**–**c**), and CB_LVX_LEU5 (**d**–**f**), TB_LVX_MAN10 (**g**–**i**), and TB_LVX_MAN5 (**j**–**l**).

SEM analysis showed that all spray-dried formulations consisted predominantly of spherical particles often exhibiting surface fractures, suggesting the presence of hollow or partially collapsed structures (Figure 2). The presence of internal cavities is consistent with gas evolution during drying, attributable to CO_2_ formation from the effervescent acid–bicarbonate system. No evident crystalline structures were observed by SEM, in agreement with typical spray-dried systems [30].

Although the morphology was broadly similar across the different formulations, some effects on particle aggregation and surface were observed depending on the type and amount of excipient used. In L-leucine-containing formulations (e.g., CB_LVX_LEU10 and CB_LVX_LEU5), a reduction in particle aggregation was observed with increasing leucine concentration. This behavior is consistent with the well-established role of L-leucine in improving powder dispersibility and reducing interparticle cohesion [27,31,32].

The surface structure of spray-dried microparticles can be interpreted using the Péclet number (Pe), defined as the ratio between the droplet evaporation rate (*κ*) and solute diffusion within the droplet (Pe = *κ*/8Di). When Pe > 1, evaporation proceeds faster than solute diffusion, leading to preferential solute accumulation at the droplet surface and the formation of a shell. Continued solvent removal induces mechanical stress, resulting in surface corrugation or partial collapse [27,33]. This mechanism is particularly relevant for L-leucine [19], which tends to precipitate early at the droplet interface, forming a surface-enriched shell [18,33].

The resulting corrugated morphology reduces interparticle contact area and contributes to the lower aggregation observed at higher leucine concentrations. Formulations containing mannitol (TB_LVX_MAN) exhibited particles with smoother surfaces and a more compact appearance, reflecting its tendency to crystallize during drying [34,35,36]. Under optimized conditions (inlet temperature 130 °C; aspiration rate 35 m^3^/h), SEM images still revealed hollow and partially fractured particles, suggesting that CO_2_ generation may contribute to internal cavity formation even in these systems.

However, the overall morphology appeared less corrugated compared to leucine-containing formulations.

From an aerodynamic perspective, a spherical morphology combined with reduced aggregation is advantageous for inhalation powders, as it generally promotes more efficient transport within the inhaled airflow and minimizes inertial deposition in the upper airways.

These morphological differences were reflected in bulk powder properties (Table 3). CB-based formulations exhibited significantly lower bulk and tapped densities compared to TB-based systems, suggesting the formation of more porous and less efficiently packed particles. This behavior is consistent with SEM observations, indicating hollow or partially collapsed structures likely associated with rapid solvent evaporation and gas generation during drying. The incorporation of L-leucine led to a slight increase in bulk density compared to the reference CB formulation, while maintaining relatively high Carr’s Index values. This suggests that, although leucine reduces interparticle cohesion (as observed from SEM), the resulting particles may still exhibit low packing efficiency due to their corrugated morphology.

In contrast, mannitol-containing formulations (TB_MAN series) showed markedly higher bulk and tapped densities, indicating the formation of denser and more compact particles. This behavior is consistent with the crystallization tendency of mannitol during spray drying, leading to structures with reduced internal porosity and improved packing ability.

To verify that the hollow and fragmented morphology is specifically attributable to CO_2_ generation, spray-dried levofloxacin without effervescent components (Levo_SD) was analyzed under identical spray drying conditions. In contrast to structures observed in the effervescent formulations, Levo_SD particles exhibited almost spherical morphology without ruptures (Figure S1).

The structural and bulk differences previously commented on translated directly into aerosol performance, evaluated using a Single Stage Glass Impinger coupled with a monodose DPI RS01 model 7 device. All formulations exhibited high ED values (>95%), indicating efficient capsule emptying and effective powder dispersion from the selected device (Table 4). These results confirm that neither the effervescent system nor the type of excipient negatively affected powder emissions.

More pronounced differences emerged in terms of FPF. Among L-leucine-containing formulations, a strong dependence on leucine concentration was evident. CB_LVX_LEU10 showed a significantly higher FPF (18.41%) compared to CB_LVX_LEU5 (2.89%), highlighting the critical role of leucine content in promoting aerosolization. This behavior is consistent with the ability of L-leucine to reduce interparticle cohesion and enhance particle dispersion [31], as also supported by SEM observations showing reduced aggregation at higher leucine concentrations.

An even more pronounced improvement in aerosol performance was observed for mannitol-containing formulations. TB_LVX_MAN10 achieved the highest FPF (27.3%), followed by TB_LVX_MAN5 (14.6%), indicating a similar concentration-dependent effect. The behavior of mannitol is consistent with its tendency to crystallize during spray drying, potentially contributing to a reduction in particle adhesion.

After evaluating the technological and aerodynamic properties of the inhalable powders, a crucial aspect of this study was the assessment of effervescent performance in terms of CO_2_ release, which directly reflects the functional capability of the system to generate mucus-perturbing gas upon contact with fluids (Table 5).

The obtained results demonstrate that CO_2_ generation in spray-dried effervescent formulations does not depend exclusively on the stoichiometry of the acid–bicarbonate reaction but is also strongly influenced by the properties of the excipients and by the particle microstructure resulting from the spray drying process. In particular, the comparison between tartaric-acid-based formulations highlighted the key role of the excipient. Despite having the same acid and bicarbonate content, the formulation containing mannitol (TB_MAN10c) exhibited significantly higher CO_2_ release than the formulation containing L-leucine (TB_LEU10). This behavior suggests that mannitol, due to its high hydrophilicity and rapid dissolution, promotes more efficient water penetration into the particles and faster contact between bicarbonate and the acidic component, thereby enhancing the completeness of the effervescent reaction. Conversely, during spray drying, L-leucine tends to migrate toward the droplet surface, forming relatively hydrophobic and corrugated outer layers. Although this behavior is advantageous for the aerosolization properties of the powder, it may delay particle hydration and consequently reduce the efficiency of CO_2_ release. The nature of organic acids also appears to contribute to the effervescent behavior of the system. The comparison between leucine-containing formulations showed higher CO_2_ release in the citric-acid-based system (CB_LEU10) than in the tartaric-acid-based formulation (TB_LEU10). This result may be associated with the triprotic nature of citric acid, which provides greater proton availability and a higher theoretical reaction capacity with bicarbonate compared with diprotic tartaric acid. The incorporation of levofloxacin resulted in a reduction in CO_2_ release in all formulations, with a particularly pronounced effect in systems containing tartaric acid and mannitol. This behavior suggests that the drug may alter the microstructure of the spray-dried matrix. Such modifications could limit effective contact between the effervescent reagents and/or promote partial entrapment of CO_2_ within the particulate matrix. Overall, the results indicate that the effervescent behavior of spray-dried formulations is governed by a complex interplay among the chemical properties of the acid, the physicochemical characteristics of the excipients, and the microstructural organization of the particles, rather than by the theoretical composition of the effervescent mixture alone.

### 3.2. Drug Dissolution and Permeation Through Mucus

To exert its therapeutic effect, the active ingredients must be released from the formulation and dissolve in the pulmonary fluids once deposited in the airways. However, the presence of a particularly dense and viscous mucus layer, as observed in conditions such as CF, can significantly hinder these processes by limiting both drug dissolution and transport.

In this study, drug release was assessed using a vertical Franz diffusion apparatus, with the powder applied either directly onto a synthetic membrane or onto a layer of porcine gastric mucus, used as a model of a viscous barrier. This approach allows a controlled comparison of the particles’ ability to penetrate a viscous medium and provides insight into the release dynamics in mucus-relevant environments.

**Figure 3:**
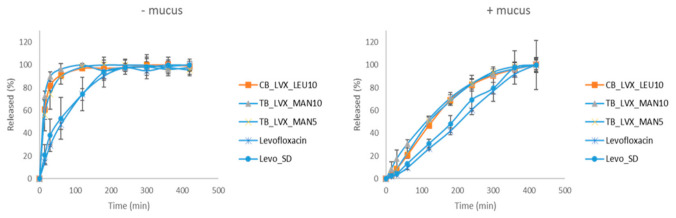
Dissolution profiles of formulations in buffer (**left**) and across a porcine mucus layer (**right**).

As a first step, the diffusion of untreated and non-respirable levofloxacin was evaluated in both conditions (Figure 3). In the absence of mucus, the drug reached near-complete release within approximately 4 h. In contrast, the presence of the mucus layer markedly reduced the amount of drug detected in the receiving compartment, confirming its role as a physical barrier that slows both dissolution and diffusion processes.

Following these preliminary experiments, spray-dried levofloxacin without effervescent components (levo_SD) was tested to isolate the contribution of particle engineering from potential effervescent effects. In both the absence and presence of mucus, levo_SD exhibited only modest improvements in drug release compared to untreated levofloxacin, suggesting that particle morphology and surface area modifications alone provide limited benefit under these conditions (detailed statistical comparisons in Tables S1 and S2).

In contrast, the full formulations containing effervescent components demonstrated substantially enhanced drug release compared to both levo_SD and raw levofloxacin. This marked improvement in release profile clearly demonstrates that the effervescent mechanism provides a distinct and essential benefit that cannot be achieved through particle engineering alone. Upon contact with the aqueous medium, the generation of carbon dioxide induces localized and transient perturbations within the surrounding environment. Although the extent of this effect cannot be directly quantified in the present model, these mechanical and physicochemical disturbances likely alter the local microstructure or hydration state of the mucus layer, thereby facilitating drug transport.

Detailed statistical comparisons for both mucus-containing and mucus-free conditions are reported in Tables S1–S6 of the Supplementary Materials.

## 4. Conclusions

The present study provides experimental evidence that effervescence significantly enhances drug release in mucus-relevant environments. Although the present work does not directly demonstrate mucus disruption, the observed behavior suggests that mild, localized effervescence could influence drug transport in mucus-like environments, supporting the potential of a “physical boost” approach based on particle engineering and controlled effervescence. However, the precise mechanisms underlying these improvements remain to be fully elucidated. Further investigations are therefore warranted to better understand the interplay between effervescence, particle behavior, and mucus microstructure. In particular, complementary approaches could include: (i) direct visualization of gas bubble formation and mucus deformation by confocal microscopy or high-speed imaging; (ii) rheological characterization of mucus exposed to the effervescent system to quantify potential changes in viscosity and elasticity; (iii) evaluation in respiratory mucus models (human or porcine tracheobronchial mucus) rather than gastric mucus, considering the compositional and structural differences relevant to cystic fibrosis lung disease; and (iv) in vivo deposition and clearance studies to assess whether the enhanced in vitro release translates into improved pulmonary bioavailability. In addition, the interplay between particle morphology, modulated by L-leucine and mannitol, and effervescent performance deserves further investigation, as these mechanisms may act synergistically. Overall, these findings provide a basis for future studies in more physiologically relevant models to better understand the impact of controlled effervescence on pulmonary drug delivery in mucus-rich conditions.

## Figures and Tables

**Table 1:** Feed composition and operating parameters of spray-dried microparticles.

Batch	Aspiration (m^3^/h)	T Inlet (°C)	Cit Out (%*w*/*v*)	Tart Out (%*w*/*v*)	Bic in (%*w*/*v*)	L-Leu * (%*w*/*w*)	Man ** (%*w*/*w*)	LVX in (%*w*/*v*)
CB	28	120	2.50	-	3.00	-	-	-
CB_LEU10	28	120	2.50	-	3.00	10.00 in 10.00 out	-	-
CB_LVX_LEU10	28	120	2.50	-	3.00	10.00 out	-	1.50
CB_LVX_LEU5	28	120	2.50	-	3.00	5.00 out	-	1.50
TB	28	120	-	2.68	3.00	-	-	-
TB_LEU10	28	120	-	2.68	3.00	10.0 in 10.0 out	-	-
TB_MAN10a	28	120	-	2.68	3.00	-	10.0 in 10.0 out	-
TB _MAN10c	35	130	-	2.68	3.00	-	10.0 in 10.0 out	-
TB_LVX_MAN10	35	130	-	2.68	3.00	-	10.0 out	1.50
TB_LVX_MAN5	35	130	-	2.68	3.00	-	5.00 out	1.50

* L-leucine to drug ratio; ** Mannitol to drug ratio.

**Table 2:** Feed composition, spray yield, and size distribution of the batches (mean ± SD, *n* = 3).

Batch	Aspiration (m^3^/h)	T Inlet (°C)	Cit Out (%*w*/*v*)	Tart Out (%*w*/*v*)	Bic in (%*w*/*v*)	L-Leu * (%*w*/*w*)	Man ** (%*w*/*w*)	LVX in (%*w*/*v*)	Yield (%)	D_50_ (µm) (SPAN)
CB	28	120	2.50	-	3.00	-	-	-	52.11 ± 2.42	9.32 ± 0.12 (1.62 ± 0.05)
CB_LEU10	28	120	2.50	-	3.00	10.0 in 10.0 out	-	-	36.52 ± 6.93	7.85 ± 0.68 (1.69 ± 0.13)
CB_LVX_LEU10	28	120	2.50	-	3.00	10.0 out	-	1.50	33.12 ± 1.21	6.28 ± 0.32 (1.76 ± 0.07)
CB_LVX_LEU5	28	120	2.50	-	3.00	5.0 out	-	1.50	35.32 ± 2.12	7.66 ± 0.54 (1.80 ± 0.87)
TB	28	120	-	2.68	3.00	-	-	-	54.20 ± 1.82	10.11 ± 0.10 (2.92 ± 0.15)
TB_LEU10	28	120	-	2.68	3.00	10.0 in 10.0 out	-	-	26.1 ± 0.83	6.35 ± 0.06 (2.01 ± 0.14)
TB_MAN10a	28	120	-	2.68	3.00	-	10.0 in 10.0 out	-	34.21 ± 1.11	-
TB _MAN10c	35	130	-	2.68	3.00	-	10.0 in 10.0 out	-	41.10 ± 1.20	9.91 ± 0.05 (2.10 ± 0.03)
TB_LVX_MAN10	35	130	-	2.68	3.00	-	10.0 out	1.50	40.13 ± 2.30	5.79 ± 0.10 (1.92 ± 0.32)
TB_LVX_MAN5	35	130	-	2.68	3.00	-	5.00 out	1.50	45.21 ± 1.71	7.64 ± 0.24 (2.19 ± 0.22)

* L-leucine to drug ratio; ** Mannitol to drug ratio.

**Table 3:** Feed composition, bulk, tapped and Carr’s index of the batches (mean ± SD, *n* = 3).

Batch	Cit Out (%*w*/*v*)	Tart Out (%*w*/*v*)	Bic in (%*w*/*v*)	L-Leu * (%*w*/*w*)	Man ** (%*w*/*w*)	LVX in (%*w*/*v*)	ρ_β_ (mg/mL)	ρ_t_ (mg/mL)	Carr’s Index (%)
CB	2.50	-	3.00	-	-	-	70.33 ± 7.50	181.64 ± 5.80	61.33 ± 3.06
CB_LEU10	2.50	-	3.00	10.0 in 10.0 out	-	-	132.23 ± 6.51	317.14 ± 15.49	58.00 ± 0.00
CB_LVX_LEU10	2.50	-	3.00	10.0 out	-	1.50	111.12 ± 0.42	247.03 ± 8.70	55.01 ± 2.01
CB_LVX_LEU5	2.50	-	3.00	5.00 out	-	1.50	84.61 ± 6.51	211.5 ± 16.26	60.00 ± 0.00
TB		2.68	3.00				99.30 ± 6.40	215.8 ± 0.61	54.01 ± 2.83
TB _MAN10c	-	2.68	3.00	-	10.0 in 10.0 out	-	208.02 ± 14.11	520 ± 35.42	70.00 ± 0.00
TB_LVX_MAN10	-	2.68	3.00	-	10.0 out	1.50	114.81 ± 1.70	280.10 ± 5.52	59.02 ± 1.41
TB_LVX_MAN5	-	2.68	3.00	-	5.00 out	1.50	105.27 ± 5.35	263.25 ± 8.44	6.120 ± 2.00

* L-leucine to drug ratio; ** Mannitol to drug ratio.

**Table 4:** Aerodynamic properties of powders (mean ± SD, *n* = 3).

Batch	Cit Out (%*w*/*v*)	Tart Out (%*w*/*v*)	Bic in (%*w*/*v*)	L-Leu * (%*w*/*w*)	Man ** (%*w*/*w*)	LVX in (%*w*/*v*)	FPF (%)	ED (%)
CB_LVX_LEU10	2.50	-	3.00	10.00 out	-	1.50	18.41 ± 2.45	97.30 ± 1.07
CB_LVX_LEU5	2.50	-	3.00	5.00 out	-	1.50	2.89 ± 1.57	100.06 ± 2.02
TB_LVX_MAN10	-	2.68	3.00	-	10.00 out	1.50	27.32 ± 3.11	99.80 ± 0.32
TB_LVX_MAN5	-	2.68	3.00	-	5.00 out	1.50	14.63 ± 2.21	95.61 ± 0.31

* L-leucine to drug ratio; ** Mannitol to drug ratio.

**Table 5:** Feed composition and average effervescence of spray-dried microparticles (mean ± SD, *n* = 3).

Batch	Cit Out (%*w*/*v*)	Tart Out (%*w*/*v*)	Bic in (%*w*/*v*)	L-Leu * (%*w*/*w*)	Man ** (%*w*/*w*)	LVX in (%*w*/*v*)	CO_2_ Released (mL)
CB	2.50	-	3.00	-	-	-	2.80 ± 0.35
CB_LEU10	2.50	-	3.00	10.00 in 10.00 out	-	-	3.78 ± 0.69
CB_LVX_LEU10	2.50	-	3.00	10.00 out	-	1.50	2.66 ± 0.30
CB_LVX_LEU5	2.50	-	3.00	5.00 out	-	1.50	2.60 ± 0.14
TB	-	2.68	3.00	-	-	-	5.52 ± 0.11
TB_LEU10	-	2.68	3.00		10.0 in 10.0 out	-	3.21 ± 0.12
TB _MAN10c	-	2.68	3.00	-	10.0 in 10.0 out	-	4.21 ± 0.02
TB_LVX_MAN10	-	2.68	3.00	-	10.0 out	1.50	2.03 ± 0.52
TB_LVX_MAN5	-	2.68	3.00	-	5.00 out	1.50	1.98 ± 0.46

* L-leucine to drug ratio; ** Mannitol to drug ratio.

## Data Availability

The original contributions presented in this study are included in the article. Further inquiries can be directed to the corresponding author.

## Supplementary Materials

The following supporting information can be downloaded at: https://www.mdpi.com/article/10.3390/pharmaceutics18070837/s1, Figure S1: SEM images of spray-dried levofloxacin without effervescent components (Levo_SD). Table S1: Statistical analysis of drug dissolution and permeation through mucus comparing raw levofloxacin and spray-dried levofloxacin without effervescent components (Levo_SD). Table S2: Statistical analysis of drug dissolution and permeation in the absence of mucus comparing raw levofloxacin and spray-dried levofloxacin without effervescent components (Levo_SD). Table S3: Statistical analysis of drug dissolution and permeation through mucus comparing raw levofloxacin and spray-dried formulations containing effervescent components (TB_LVX_MAN10, TB_LVX_MAN5, and CB_LVX_LEU10). Table S4: Statistical analysis of drug dissolution and permeation through mucus comparing spray-dried levofloxacin without effervescent components (Levo_SD) and spray-dried formulations containing effervescent components (TB_LVX_MAN10, TB_LVX_MAN5, and CB_LVX_LEU10). Table S5. Statistical analysis of drug dissolution and permeation in the absence of mucus comparing raw levofloxacin and spray-dried formulations containing effervescent components (TB_LVX_MAN10, TB_LVX_MAN5, and CB_LVX_LEU10). Table S6: Statistical analysis of drug dissolution and permeation in the absence of mucus comparing spray-dried levofloxacin without effervescent components (Levo_SD) and spray-dried formulations containing effervescent components (TB_LVX_MAN10, TB_LVX_MAN5, and CB_LVX_LEU10).

## Abbreviations

The following abbreviations are used in this manuscript:

Bic	Sodium Bicarbonate
CF	Cystic Fibrosis
Cit	Citric Acid
CO_2_	Carbon Dioxide
DPI	Dry Powder Inhaler
ED	Emitted Dose
FPF	Fine Particle Fraction
L-Leu	L-Leucine
LVX	Levofloxacin
Man	Mannitol
SEM	Scanning Electron Microscopy
SSGI	Single Stage Glass Impinger
Tart	Tartaric Acid
UV	Ultraviolet
