# Supplementary material for: Design and Production of Respirable Effervescent Microparticles to Enhance Drug Penetration Through Lung Mucus
Source: Pharmaceutics. 2026 Jul 9;18(7):837. doi: 10.3390/pharmaceutics18070837 (PMC13414476; doi:10.3390/pharmaceutics18070837)
Supplement: Supplementary file 1 [file pharmaceutics-18-00837-s001.zip › pharmaceutics-4316830-supplementary.pdf]

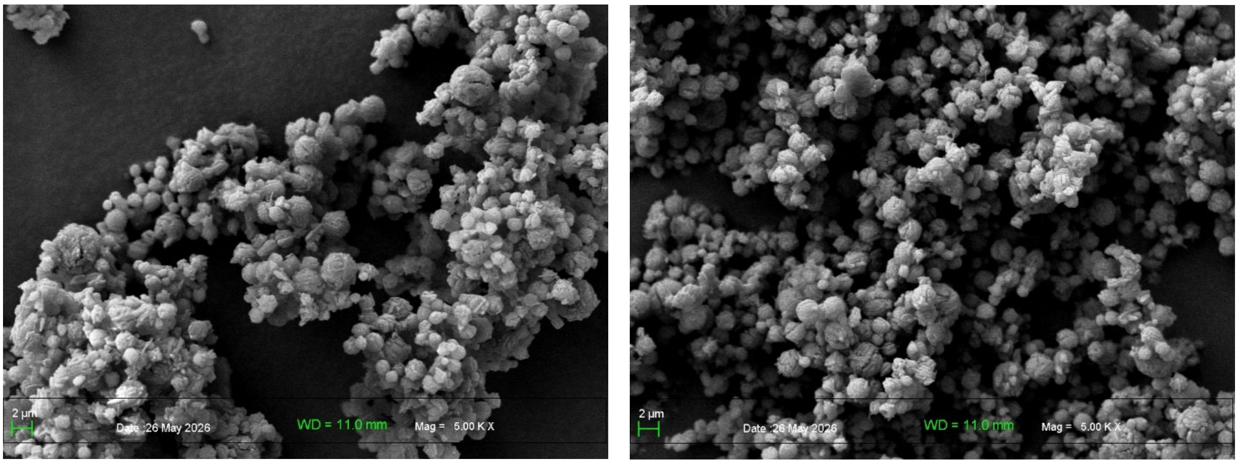

Figure S1. SEM images of spray-dried levofloxacin without effervescent components (Levo_SD). Levo_SD particles exhibit intact, spherical morphology with minimal surface irregularities.

Table S1. Statistical analysis of drug dissolution and permeation through mucus comparing raw levofloxacin and spray-dried levofloxacin without effervescent components (Levo_SD). Statistical comparisons were performed at each sampling time point (15, 30, 60, 120, 180, 240, 300, 360, and 420 min) using one-way ANOVA followed by Bonferroni's multiple comparison post hoc test. Statistical significance is indicated as follows: $p < 0.05$ (*), $p < 0.01$ 0(**), $p < 0.001$ (***), and $p < 0.0001$ (****).

Comparison	15	30	60	120	180	240	300	360	420
Levofloxacin vs. Levo_SD	-	-	-	-	-	-	-	-	-

Table S2. Statistical analysis of drug dissolution and permeation in the absence of mucus comparing raw levofloxacin and spray-dried levofloxacin without effervescent components (Levo_SD). Statistical comparisons were performed at each sampling time point (15, 30, 60, 120, 180, 240, 300, 360, and 420 min) using one-way ANOVA followed by Bonferroni's multiple comparison post hoc test. Statistical significance is indicated as follows: $p < 0.05$ (*), $p < 0.01$ (**), $p < 0.001$ (***), and $p < 0.0001$ (****).

Comparison	15	30	60	120	180	240	300	360	420
Levofloxacin vs. Levo_SD	-	-	-	-	-	-	-	-	-

Table S3. Statistical analysis of drug dissolution and permeation through mucus comparing raw levofloxacin and spray-dried formulations containing effervescent components (TB_LVX_MAN10, TB_LVX_MAN5, and CB_LVX_LEU10). Statistical comparisons were performed at each sampling time point (15, 30, 60, 120, 180, 240, 300, 360, and 420 min) using one-way ANOVA followed by Bonferroni's multiple comparison post hoc test. Statistical significance is indicated as follows: $p < 0.05$ (*), $p < 0.01$ (**), $p < 0.001$ (***), and $p < 0.0001$ (****).

Comparison	15	30	60	120	180	240	300	360	420
Levofloxacin vs. TB_LVX_MAN10	-	***	****	****	****	****	****	-	-
Levofloxacin vs. TB_LVX_MAN5	-	-	***	****	****	****	****	-	-
Levofloxacin vs. CB_LVX_LEU10	-	-	**	****	****	****	**	-	-

Table S4. Statistical analysis of drug dissolution and permeation through mucus comparing spray-dried levofloxacin without effervescent components (Levo_SD) and spray-dried formulations containing effervescent components (TB_LVX_MAN10, TB_LVX_MAN5, and CB_LVX_LEU10). Statistical comparisons were performed at each sampling time point (15, 30, 60, 120, 180, 240, 300, 360, and 420 min) using one-way ANOVA followed by Bonferroni's multiple comparison post hoc test. Statistical significance is indicated as follows: $p < 0.05$ (*), $p < 0.01$ (**), $p < 0.001$ (***), and $p < 0.0001$ (****).

Comparison	15	30	60	120	180	240	300	360	420
Levo_SD vs. TB_LVX_MAN10	-	*	**	***	***	*	-	-	-
Levo_SD vs. TB_LVX_MAN5	-	-	-	**	***	*	*	-	-
Levo_SD vs. CB_LVX_LEU10	-	-	-	**	***	*	-	-	-

Table S5. Statistical analysis of drug dissolution and permeation in the absence of mucus comparing raw levofloxacin and spray-dried formulations containing effervescent components (TB_LVX_MAN10, TB_LVX_MAN5, and CB_LVX_LEU10). Statistical comparisons were performed at each sampling time point (15, 30, 60, 120, 180, 240, 300, 360, and 420 min) using one-way ANOVA followed by Bonferroni's multiple comparison post hoc test. Statistical significance is indicated as follows: $p < 0.05$ (*), $p < 0.01$ (**), $p < 0.001$ (***), and $p < 0.0001$ (****).

Comparison	15	30	60	120	180	240	300	360	420
Levofloxacin vs. TB_LVX_MAN10	****	****	****	****	-	-	-	-	-
Levofloxacin vs. TB_LVX_MAN5	****	****	****	****	-	-	-	-	-
Levofloxacin vs. CB_LVX_LEU10	****	****	****	****	-	-	-	-	-

Table S6. Statistical analysis of drug dissolution and permeation in the absence of mucus comparing spray-dried levofloxacin without effervescent components (Levo_SD) and spray-dried formulations containing effervescent components (TB_LVX_MAN10, TB_LVX_MAN5, and CB_LVX_LEU10). Statistical comparisons were performed at each sampling time point (15, 30, 60, 120, 180, 240, 300, 360, and 420 min) using one-way ANOVA followed by Bonferroni's multiple comparison post hoc test. Statistical significance is indicated as follows: $p < 0.05$ (*), $p < 0.01$ (**), $p < 0.001$ (***), and $p < 0.0001$ (****).

Comparison	15	30	60	120	180	240	300	360	420
Levo_SD vs. TB_LVX_MAN10	****	****	****	***	-	-	-	-	-
Levo_SD vs. TB_LVX_MAN5	****	****	****	***	-	-	-	-	-
Levo_SD vs. CB_LVX_LEU10	****	****	****	**	-	-	-	-	-
